# Object based classification of a riparian environment using ultra-high resolution imagery, hierarchical landcover structures, and image texture

**DOI:** 10.1038/s41598-022-14757-y

**Published:** 2022-07-04

**Authors:** Kain Kutz, Zachary Cook, Marc Linderman

**Affiliations:** 1 United States Department of Agriculture Forest Service, 125 South State Street, Suite 7105, Salt Lake City, UT 84138 USA; 2 Department of Geographical and Sustainability Sciences, University of Iowa, 316 Jessup Hall, Iowa, IA 52242 USA

**Keywords:** Plant ecology, Structural geology, Geology, Sedimentology, Behavioural ecology, Biogeography, Community ecology, Ecological modelling, Ecological networks, Ecosystem ecology, Freshwater ecology, Riparian ecology, Wetlands ecology, Information technology, Biotechnology, Computational biology and bioinformatics, Ecology, Plant sciences, Environmental sciences, Hydrology, Limnology, Mathematics and computing

## Abstract

Land cover mapping is an important part of resource management, planning, and economic predictions. Improvements in remote sensing, machine learning, image processing, and object based image analysis (OBIA) has made the process of identifying land cover types increasingly faster and reliable but these advances have not been able to utilize all of the information encompassed within ultra-high (sub-meter) resolution imagery. There have been few known attempts to try and maximize this detailed information in high resolution imagery using advanced textural components. Hierarchical land classes are also rarely used as an attribute within the machine learning step of object-based image analysis. In this study we try to circumnavigate the inherent problems associated with high resolution imagery by combining well researched data transformations that aid the OBIA process with a seldom used texture transformation in Geographic Object Based Image Analyses (GEOBIA/OBIA) known as the Gabor Transform and the hierarchal organization of landscapes. We will observe the difference made in segmentation and classification accuracy of a random forest classifier when we fuse a Gabor transformed image to a Normalized Difference Vegetation Index (NDVI), high resolution multi-spectral imagery (RGB and NIR) and Light Detection and Ranging (LiDAR) derived canopy height model (CHM) within a riparian area in Southeast Iowa, United States. Additionally, we will observe the effects on classification accuracy when adding multi-scale land cover data to objects. Both, the addition of hierarchical information and Gabor textural information, could aid the GEOBIA process in delineating and classifying the same objects that human experts would delineate within this riparian landscape.

## Introduction

Remote sensing has played a critical role in the development of the science of landscape ecology^1^. Satellite and aerial imagery allow the quantification not only of the composition, or amounts of different land covers, of a landscape, but also the spatial structure or arrangement of land cover as well. Visual interpretation of high-resolution imagery has been crucial in the delineation and verification of land cover, particularly in complex ecosystems. Automated approaches to classifying imagery, such as Geographic Object Based Image Analysis (GEOBIA), is increasingly being used to assess historical aerial, UAV, and high-resolution limited-spectral satellite data^2–4^. However, its performance varies across different landscapes. For example, in most object based image analyses of urban areas, classification accuracy is above 90%^3,5–9^ while within a natural multipart landscape, with little human influence, it is expected that the accuracy will be well below 90%^5,10–14^. The goal of this study is to examine the use of hierarchical and image transformations to object delineation and classification in complex natural landscapes.

GEOBIA replicates the process of human object recognition using spatial information by first creating individual polygons or objects (segmentation). Statistics about these objects, such as edge complexity and spectral variance, are then used to determine which class the object belongs (classification). Utilizing the natural hierarchical organization within ecosystems could provide additional information which improves classification accuracies. The primary instances of developing a hierarchical scheme into the GEOBIA process is to reduce segmentation errors by decreasing noise or attempting to increase classification accuracy using either a rule-based classifier or fuzzy classifier^15–20^. This approach does not leverage the information supported by landscape hierarchy theory, a framework for scaling and understanding the relationship between spatial pattern and ecological process.

Within landscape ecology, O’Neill et al.^21^ conducted a meta-analysis of hierarchal frameworks in biology. They concluded that the various scales within an ecological system define, or limit, one another in a way that could support that a super-object could be a useful property in defining sub-objects within a landscape. If multiple classifications are performed at several scales, the attributes of larger scaled objects (i.e. super-objects) can be tied to the smaller scaled objects (i.e. sub-objects), thus potentially increasing the classification accuracy of the sub-objects. This approach uses what we know about the organization of complex multipart ecosystems. However, remote sensing analytical techniques, such as GEOBIA, have not incorporated hierarchical landscape ecology theory in classification methodologies nearly as in depth. The primary use of hierarchical landscape organization in OBIA is the iterative process of classifying a landscape into sub-classes from super classes. An example of this approach can be found in Mao et al.^22^. In their paper they first classified their segmented image into wetland/non-wetland classes and then classified the wetland objects into smaller and smaller subclasses using thresholds or rule-based classifiers.

As opposed to dissecting classified objects into smaller subclasses, in this paper we use the information from a separate classification that uses a higher-level class schema to contribute to the classification of smaller sub-objects that uses a lower-level class schema. This allows us to examine the role of hierarchical information inherent in natural landscapes and image processing techniques to better develop automated replication of visual interpretation of natural landscapes. Specifically, we examine the impact of hierarchical segmentation on object classification, relative to a multi-scale visual classification, of high-resolution aerial imagery.

Segmenting images into hierarchical objects consistent with visual delineation could also be enhanced by image enhancement that is consistent with human interpretation. The Gabor textural transformation has been lauded for replicating the same directional textural information that humans use to identify and interpret objects^23–25^. However, few studies have been conducted that investigate the use of this transformation for object-based image analysis^26,27^.

We aim to examine the accuracy improvement from hierarchical delineation and classification of complex floodplains by combining well-researched data transformations, that aid the OBIA process, with a seldom-used texture transformation in GEOBIA known as the Gabor Transform. We used a random forest classifier, three band (near-infrared, red, and green) 7.9-cm imagery, Normalized Difference Vegetation Index (NDVI) and a Light Detection and Ranging (LiDAR) derived canopy height model (CHM) within a riparian area in Southeast Iowa; allowing us to observe the difference in segmentation and classification accuracy that a Gabor transform and hierarchical land cover data can provide to object based analysis.

## Data and study area

### Data

The aerial imagery, used for our study, is a three band (near-infrared, red, and green) 7.9-cm resolution image taken with an Applanix 439 Digital Sensor System on May 18, 2014. The images were taken by the U.S. Fish & Wildlife Service, Region 3, and the U.S. Geological Survey’s Upper Midwest Environmental Sciences Center. The CHM used in this paper is from the Iowa LiDAR Project^28^. LiDAR data was downloaded as several four-square kilometer, las tiles that encompassed the study area and was originally collected on May 5, 2010. The files were converted into a last-return digital terrain model (DTM) TIFF files using the ArcGIS Lidar Analyst Extension. The CHM was then created by subtracting the DTM from a digital surface model (DSM) derived from the first return values. All imagery and vector files were projected and processed within the Universal Transverse Mercator zone 15 spatial reference. All sets of data were collected during leaf-on conditions. Reference polygons were hand delineated and classified by experts from US Fish and Wildlife Service Region 3, Port Louisa National Wildlife Refuge, and the USGS Upper Midwest Environment Sciences Center. This data allowed us to perform a two-tier classification as the visual classification used two object classification schemas; a broad 7-class scheme and a narrower 13-class scheme. Using these schemas to train and base our classifications upon, we examined the improvement in classification accuracy of floodplain sub-objects.

### Study area

**Figure 1:**
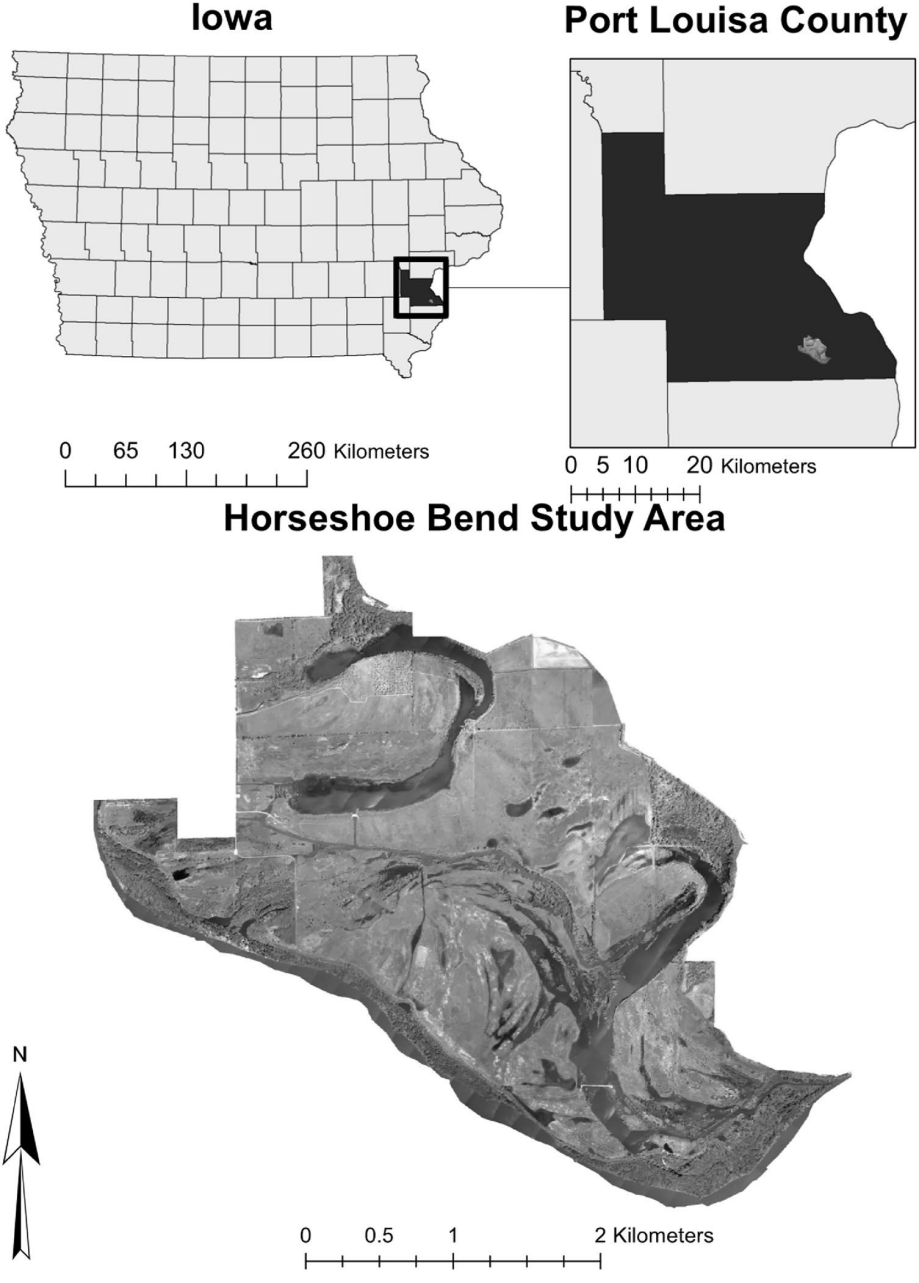
Horseshoe bend division of the Port Louisa NWR (study area). Software: ArcMap (10.x).

The Horseshoe Bend Division of the Port Louisa National Wildlife Refuge (NWR) is a mixture of grass and wetland habitat along the Iowa River four miles upstream from the confluence of the Iowa and Mississippi River. This 2606-acre NWR is composed of grassland, wet meadows, forest, and semi and permanently flood emergent wetland habitat. Prior to the 1993 flood, this land was primarily used for agricultural purposes and was protected from flooding by a levee along the Iowa River. Since then, the levee has broken along the upper reach where the Iowa River intersects the NWR making the land susceptible to frequent inundation. This study area is in Port Louisa County Southeast of Wapello, Iowa (see Fig. 1).

## Methodology

### Gabor transform

The Gabor transform has rarely been used as a feature in a landscape classification OBIA approach but has been used in other OBIA processes such as fingerprint enhancement and human iris detection and for data dimensionality reduction^24,29–35^. Gabor filters are a bandpass filter applied to an image to identify texture. The different Gabor bandpass filters mathematically model the visual cortical cells of mammalian brains and thus is expected to improve segmentation and classification accuracy when compared to a human delineated and classified image^26,27^.

Samiappan et al.^36^ compared Gabor filters to other texture features (grey-level co-occurrence matrix, segmentation-based fractal texture analysis, and wavelet texture analysis) within the GEOBIA process, of a wetland, using sub-meter resolution multispectral imagery. These Gabor filters performed comparably, in overall classification accuracy and Kappa coefficients, with other texture features. However, they were still outperformed by all other texture features. This study did not use any other data for analysis for determining the performance of Gabor filters when paired with data sources such as spectral, NDVI, or LiDAR^36,37^. Wang et al.^38^ paired a Gabor transformation with a fast Fourier transformation for edge detection on an urban landscape image that contained uniform textures with promising results. Su^30^ used the textural attributes derived from Gabor filters for classification but had similar results to Samiappan et al.^36^ where they found that Gabor features were one of the least useful/influential that contributed to the classification of a mostly agricultural landscape.

Gabor filters are a Fourier influenced wavelet transformation, or bandpass filter, that identifies texture as intervals in a 2-D Gaussian modulated sinusoidal wave. This modulation differentiates the Gabor transform from the Fourier transform^23,26^. These Gabor transformed wavelets are parameterized by the angle at which they alter the image and the frequency of the wavelet. Rather than smoothing an image at the cost of losing detail through Fourier transforms or median filters, Gabor transformed images identify the repeated pattern of localized pixels and gives them similar values if they are a part of the same repeated sequence. Gabor features can closely emulate the visual cortex of mammalian brains that utilize texture to identify objects^26,27^. This is based on the evaluation of neurons associated with the cortical vertex that respond to different images or light profiles^39^. Marcelja^27^ identified that cortical cells responded to signals that are localized frequencies of light like what is represented by the Gabor transformations. Within the frequency domain, the Gabor transform can be defined by Eq. (1):

1 $$G\left(u, v;f, \theta \right)= {e}^{-\frac{{\pi }^{2}}{{f}^{2}} ({\gamma }^{2}({u}^{{\prime}}-f{)}^{2}+{n}^{2}{v}^{{{\prime}}2})}$$

where $f$ is the user-determined frequency (or wavelength); $\theta$ is the user-determined orientation at which the wavelet is applied to the image; $\gamma$ and $n$ are the standard deviations of the Gaussian function in either direction^23,38^. These parameters define the shape of the band pass filter and determines its effect on one-dimensional signals. Daugman^26^, created a 2-D application of this filter in Eq. (2);

2 $$g\left(u,v\right)= {e}^{-{\pi }^{2}/{f}^{2}[{\gamma }^{2}{\left({u}^{{\prime}}-f\right)}^{2}+{n}^{2}{{v}^{{\prime}}}^{2}]}$$

where u' = ucos − vsin θ θ and v' = usin − vcos θ.

In order to implement Gabor filters on multi-band spectral images, we used Matlab’s Gabor feature on the University of Iowa’s Neon high performance computer (HPC)^40^ which has up to 512 GB of RAM, which was necessary for processing these images. The first implementation of Gabor filters was performed on a 1610 × 687 single band pixel array (a small subset of the study area), a filter bank of 4 orientations and 8 wavelengths, on a 32 GB RAM computer, and took approximately 8 h to complete. Filter banks are a set of Gabor filters with different parameters that is applied to the spectral image and are required to identify different textures with different orientations and frequencies. By lowering the number of wavelengths from 8 to 4 on an 8128 × 8128 single band pixel array on the same machine 32 GB RAM, the processing was reduced to an hour. Using the HPC, this was further reduced to approximately 90 s using the same filter bank. Before implementing on the HPC, the original spectral image was divided into manageable subsets with overlap in order to prevent ‘edge-effect.’ These images were converted to greyscale by averaging values across all three bands^33^. When wavelengths become too long, they no longer attribute the textural information desired from the image and therefore add unnecessary computing time. The wavelengths that were used for the filter bank were selected as increasing powers of two starting from 2.82842712475 ($24/\sqrt{2}$) up to the pixel length of the hypotenuse of the input image. From this, we used only 2.82842712475, 7.0710678, 17.6776695, and 44.19417382. The directional orientation was selected as 45° intervals, from 0 to 180: 0, 45, 90, 135. These parameters were based on the reasoning outlined within Jain and Farrokhina^25^. More directional orientations could have been included but four were used for computational efficiency. The radial frequencies were selected so that they could capture the different texture in the landscape represented by consistent changes in pixels values within each landcover class. When frequencies are too wide or fine of a width they no longer represent the textures of the different landcover classes and thus are not included. This selection of filter bank parameters are similar or the same as other studies that look into the use of Gabor features for OBIA^25,30,31^.

**Figure 2:**
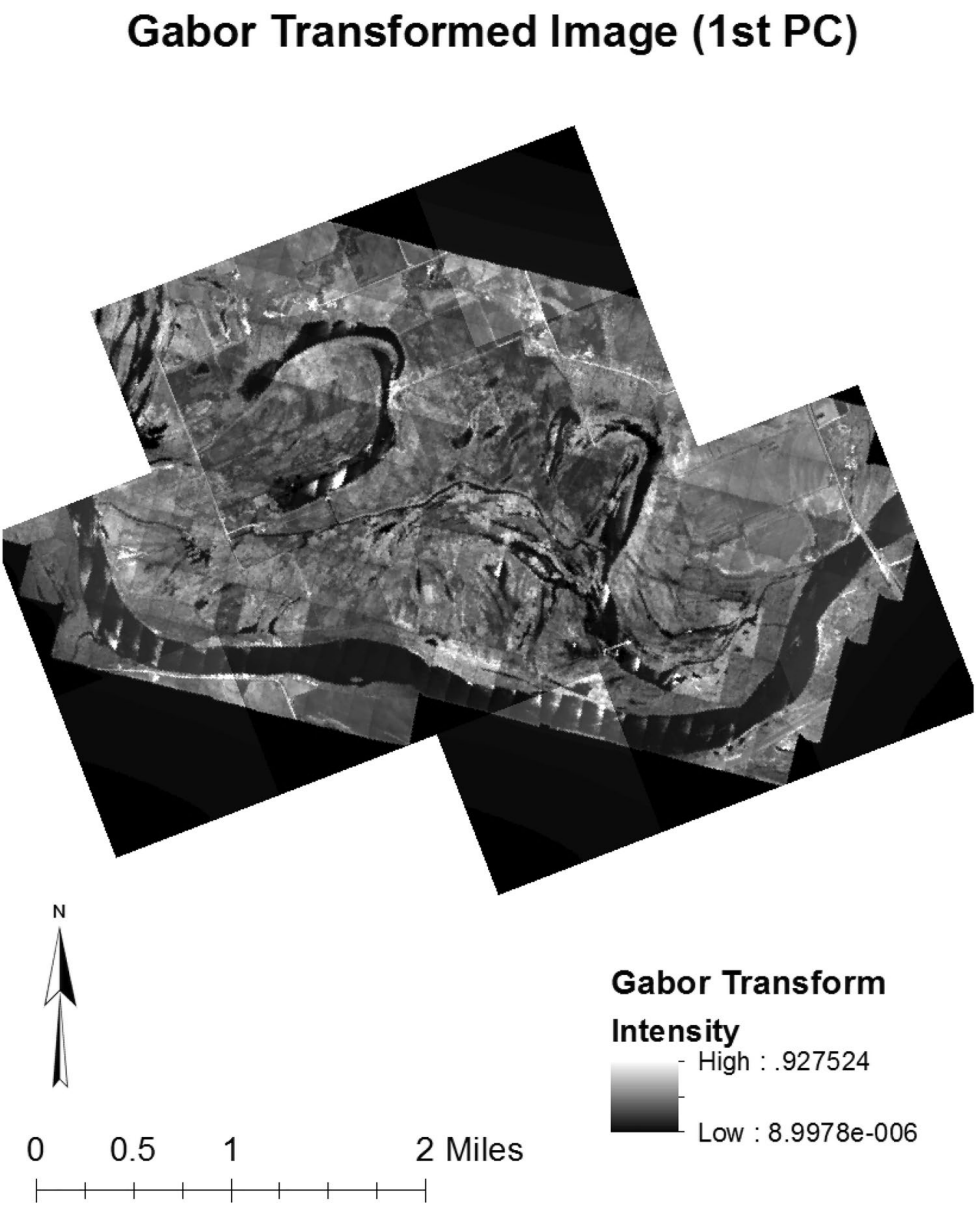
Gabor transformation. Gabor transformed image of study area derived from original image using the first principal component of all gabor outputs using the filter bank parameters. Software: ArcMap (10.x).

From the different combinations of parameters (four directions and four frequencies) in the Gabor Transform filter bank, sixteen magnitude response images were created from the converted greyscale three band average image. To limit high local variance within the output Gabor texture images, a Gaussian filter was applied. The magnitude response values were normalized across the 16 different bands so that a Principal Component Analysis (PCA) could be applied. The first principal component of the PCA, from these Gabor transformed images, was used for this study since it limits the computation time to process 16 separate Gabor features, in addition to the other data sources, while still retaining the most amount of information from the different Gabor response features. The Gabor band that was used for this study can be viewed in Fig. 2.

### Segmentation

For this study, we used the watershed algorithm for the segmentation of GEOBIA, implemented by ENVI version 5.0 Feature Extraction tool, due to its ubiquitous use within GEOBIA, its ability to create a hierarchy of segmented objects, and support within the literature as a reliable algorithm^37,41–43^. The watershed algorithm can either use a gradient image or intensity image for segmentation. Based on the observed results, this study used the intensity method. The intensity method averages the value of pixels across bands. Scale, a user-defined parameter, is selected to identify the threshold that decides if a given intensity value within the gradient image can be a boundary. This allows the user to decide the size of the objects created. A secondary, user-defined, parameter defines how similar, adjacent, objects need to be before they are combined or merged. The user arbitrarily selects the parameter value based on how it reduces both under and over segmentation. The parameters selected for this study were visually chosen based on a compromise between over and under segmentation relative to the hand demarcated objects.

The merging of two separate objects was based on the full lambda schedule where the user selects a merging threshold ${t}_{i, j}$ which is defined by Eq. (3):

3 $${t}_{i, j}= \frac{\frac{\left|{O}_{i}\right|\cdot \left|{O}_{j}\right|}{\left|{O}_{i}\right|+ \left|{O}_{j}\right|}\cdot {\Vert {u}_{i}-{u}_{j}\Vert }^{2}}{\mathrm{length}(\mathrm{\vartheta }\left({O}_{i},{O}_{j}\right))}$$

where ${O}_{i}$ is the object of the image, $\left|{O}_{i}\right|$ is the area of $i$, ${u}_{i}$ is the average of object $i$, ${u}_{j}$ is the average of object $j$, $\Vert {u}_{i}-{u}_{j}\Vert$ is the Euclidean distance between the average values of the pixel values in regions $i$ and $j$, and $\mathrm{length}\left(\mathrm{\vartheta }\left({O}_{i},{O}_{j}\right)\right)$ is the length of the shared boundary of ${O}_{i}$ and ${O}_{j}$.

**Figure 3:**
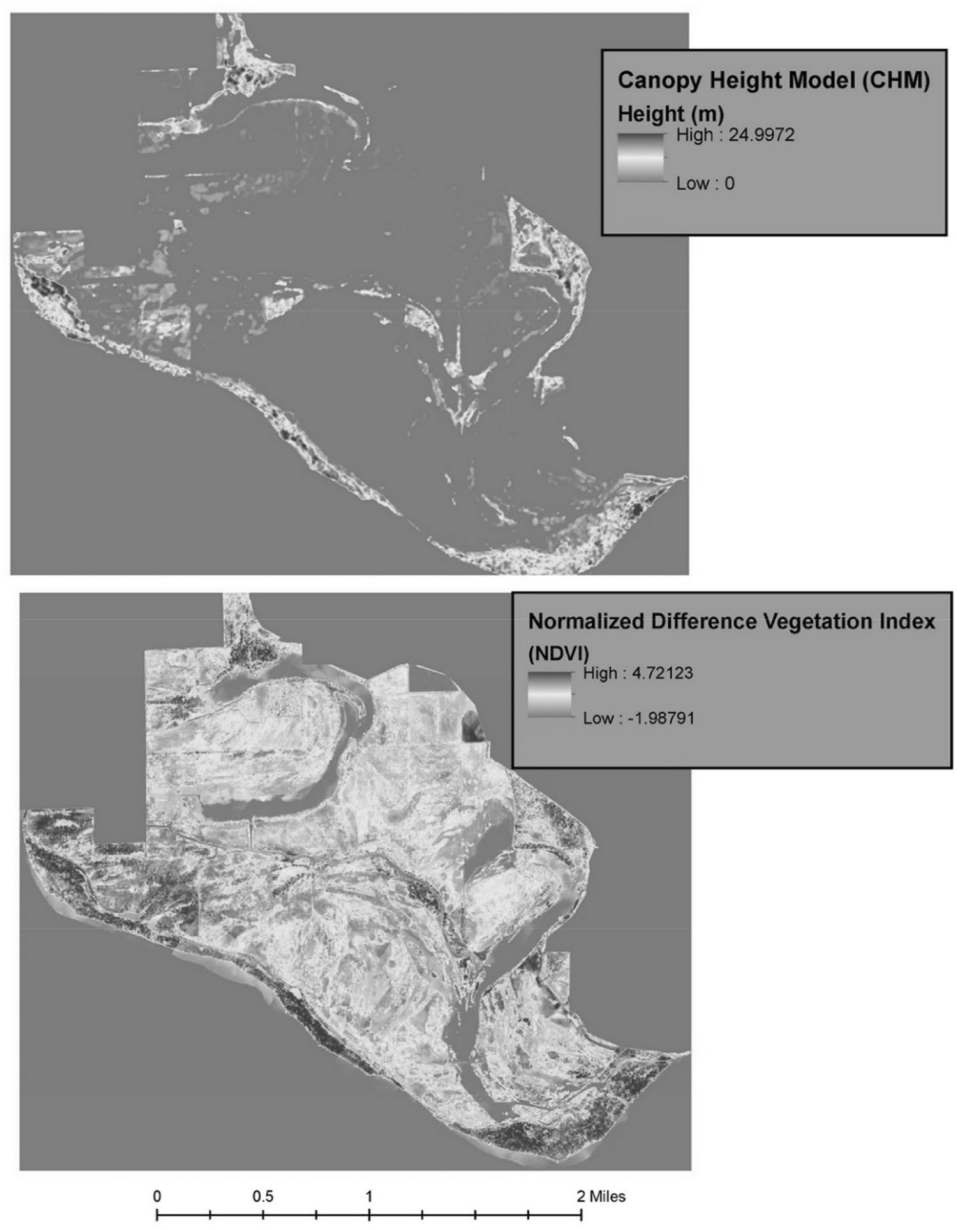
CHM and NDVI. LiDAR derived canopy height model (top) and normalized difference vegetation index derived from original spectral image. Software: ArcMap (10.x).

To compare the segmentation of a riparian landscape, with and without Gabor features, we conducted segmentation on two separate sets of data. One dataset was a normalized stacked layer of NDVI and CHM (see Fig. 3) with the original multispectral image used as ancillary data; the other dataset differed only by the inclusion of the Gabor feature. For both instances, the bands were converted to an intensity image by averaging across bands rather than being converted into a gradient image for segmentation. The dataset that included the Gabor features had a scale parameter set at 30 with merge settings at 95 and 95.7 for the sub and super-objects, respectively. The dataset that did not include the Gabor features had a scale parameter of 10 with merge settings at 95.6 and 98.5 for the sub and super-objects, respectively. This resulted in the creation of 87,198 and 62,905 segments for the sub and super objects, respectively, that were created when the Gabor feature was included. 191,050 and 51,664 segments were created for the sub and super objects when the Gabor features, respectively, were not included within the segmentation process. As you will see in the next section, these segments also represent the number of training data that will be included within the supervised classification.

**Figure 4:**
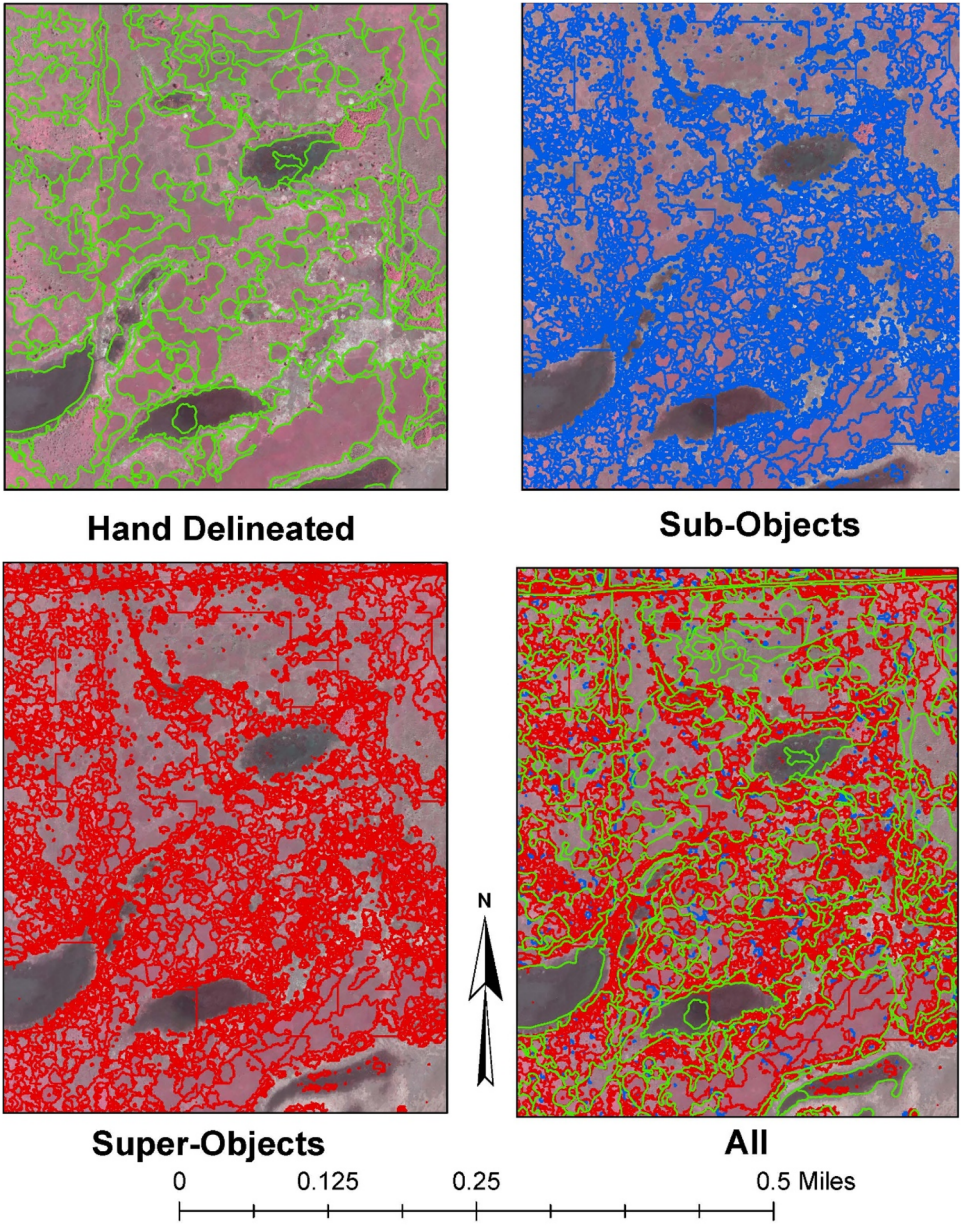
Automated and manual segmented comparison. Juxtaposition of hand delineated, sub-objects, and super-objects for segments generated using the Gabor features. Software: ArcMap (10.x).

To create a hierarchy of land cover classes, two sets of segmentation parameters needed to be selected for each dataset. One set of parameters would be used for the sub-objects within the hierarchy and the other set would be used to create super-objects. All parameters used the intensity and full lambda schedule algorithms for the watershed method. The only setting that changed between the sub and super-objects, for either dataset, was the merge parameter which helped maintain similar boundaries as much as possible. Despite this, boundaries could moderately change due to the Euclidean distance, between the pixel values of $i$ and $j$, changing from the merging of objects; causing ${t}_{i, j}$ to cross the threshold which results in a new boundary being drawn. A representation of these results can be viewed and visually compared to the hand demarcated objects in Fig. 4.

### Training data

**Figure 5:**
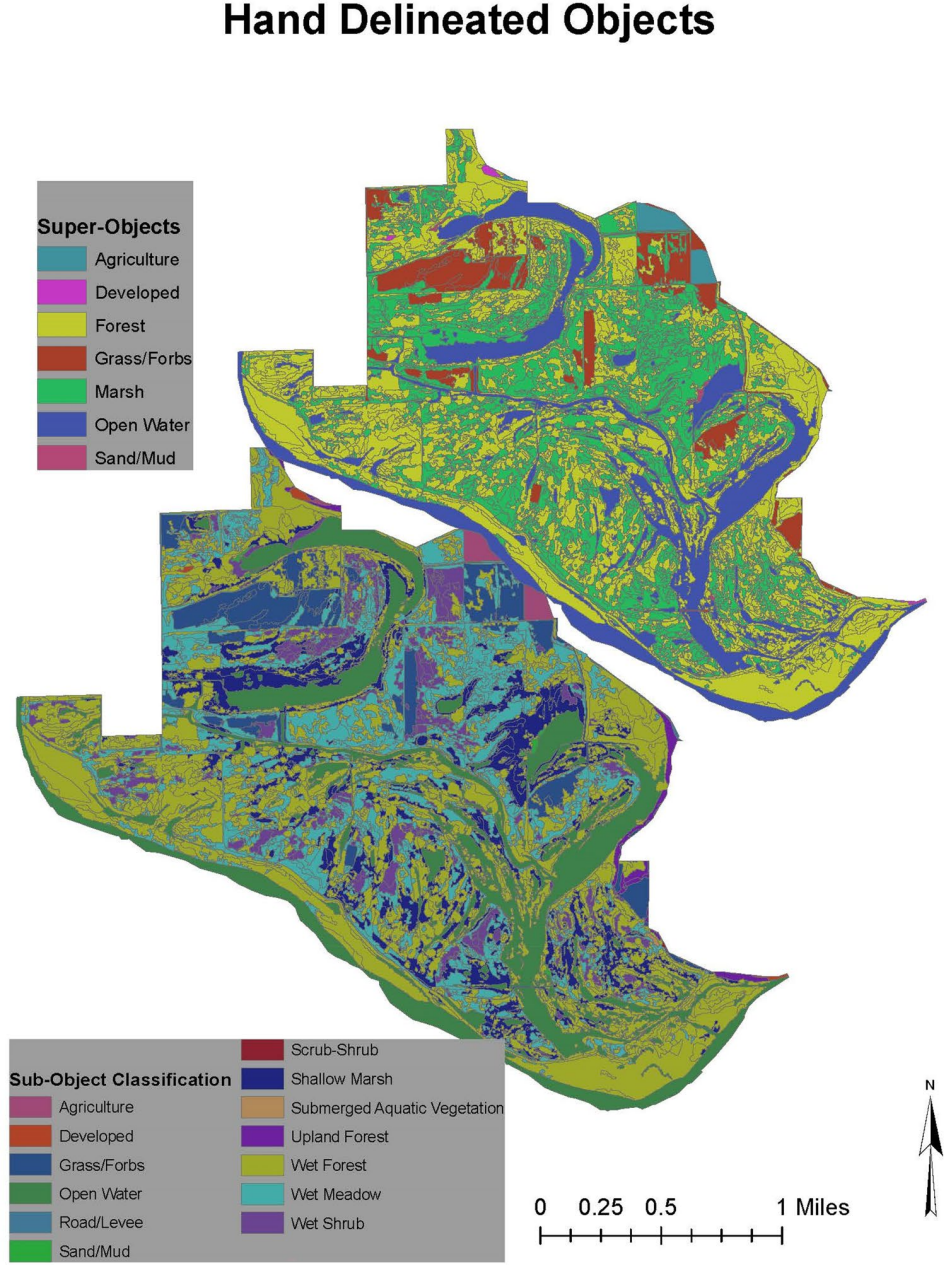
Hand delineated objects of both scales. Software: ArcMap (10.x).

The training data, used for this study, is the transfer of class attributes from hand demarcated and classified segments to automatically segmented objects based on the majority overlap of the hand demarcated segments. Experts identified them using two different classification schemes referenced from the General Wetland Vegetation Classification System^44^. The 7-class scheme within this system identified objects of either being forest, marsh, agriculture, developed, open water, grass/forbs, or sand/mud. The 13-class scheme identified objects of either being agriculture, developed, grass/forbs, open water, road/levee, sand/mud, scrub-shrub, shallow marsh, submerged aquatic vegetation, upland forest, wet forest, wet meadow, and wet shrub. Not every class from the 7-class scheme will have a sub-class (i.e. developed, open water) but some do for example wet and upland forest are sub-objects of the forest class and wet meadow and shallow marsh are sub-objects of marsh. Figure 5 visually illustrates both classification schemes across the study area.

ENVI’s feature extraction tool calculates several landscape, spectral, and textural metrics. These attributes were used for each random forest classifier. The Gabor and Hierarchical features will be included selectively to be able to compare their contributions to the (out-of-bag) OOB classification errors. When Gabor features are included within the classification, they are computed the same way as the other image bands.

### Random forest

The random forest classifier was implemented in R using the random forest module^45^. The number of trees, that were randomly generated, was large enough (n = 250) to where the Strong law of large numbers would take effect as indicated by the decrease in the change of accuracy. The default number of variables randomly sampled as candidates at each split variable (mtry parameter) was the total number of variables divided by 3 for each dataset. R also generates two separate variable indices: mean decrease in accuracy and mean decrease Gini. Mean decrease in accuracy refers to the accuracy change in the random forest when a single variable is left out. This is a practical metric to determine the usefulness of a variable. The Gini index measures the purity change within a dataset when it is split based upon a given variable within a decision tree.

The random forest classification accuracy will be based on the OOB error. The random forest algorithm trains numerous decision trees on random subsets of the training set leaving out a number of training samples when training each decision tree. The samples that are left out of each decision tree are then classified by the decision tree that they were not included within during the training step. The OOB error is the average error of each predicted bootstrapped sample across the ensemble of decision trees within the random forest algorithm.

**Figure 6:**
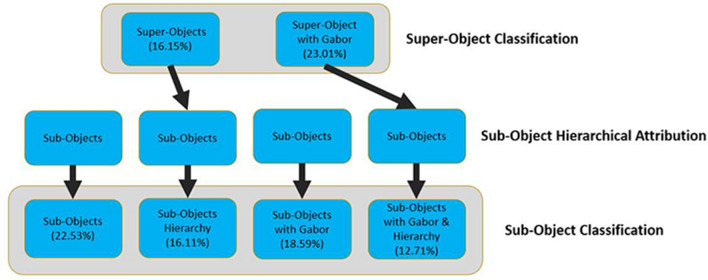
Classification procedure. Schematic flow chart illustrating how the Gabor and hierarchal features were included within the classification of the super and sub-objects. OOB classification error included in parenthesis.

Figure 6 illustrates how the Gabor and hierarchal features were included within the classification of the super and sub-objects.

### Hierarchical scheme

To attribute the hierarchical structure to the sub-objects, we first classified the larger segments that were created with and without the Gabor features using the broader 7-class scheme. These classified super objects were then converted to raster to calculate the majority overlap with the smaller sub-objects. This gave the sub-objects an attribute, the broader 7-class scheme, that could be used to contribute to the classification of the sub-objects with the finer 13-class scheme. This builds the hierarchical relationship between the two class schemes into the supervised classification of the sub-objects. Figure 6 illustrates how the hierarchal structure was included within two of the four sub-object’s list of features used within classification. This methodological approach aligns with O’Neill et al.^21^ landscape ecology principle that a super-object’s class could be a useful property in defining or predicting a sub-object. This is also different than the more common rule-based approach of iteratively classifying the landscape into smaller and smaller sub-classes^22^.

### Segmentation assessment

Most studies rely upon the accuracy assessment of their classifiers to provide support for their analysis results. However, this does not provide evidence whether a new data fusion technique improves the ability to delineate objects of interest within an image. To assess the performance of our segmented polygons, this study evaluated the segments created with and without the Gabor feature using a method highlighted in Xiao et al.^37^.

Our segmentation results were evaluated using an empirical discrepancy measure, used frequently in image segmentation evaluation^37,46,47^. Discrepancy measures utilize ground truth images that represent the “correct” delineated/classified image to compare the semi-automated image results. In our study, the objects that were delineated and classified by experts from the U.S. Fish and Wildlife Service, were used as training data for our random forest classifier and as ground truth for the discrepancy measure. The discrepancy measure used the percentage of right segmented pixels (PR) in the whole image. To calculate PR, we converted the classified segmented and ground truth polygons to raster and measured the ratio of incorrect pixels to total amount of pixels which was converted to a percentage.

Additionally, landscape metrics were calculated using FRAGSTATS^48^, an open source program commonly used for calculating landscape metrics. FRAGSTATS computed these metrics from thematic raster maps that represent the land cover types of interest. These thematic classes, used for analysis, were the classified objects at both the super and sub-object level. Since we are not attempting to compare the segmentation results for any specific class or area, we calculated metrics on a landscape level. Landscape metrics will represent the segmentation patterns for the entire study area.

FRAGSTATS can calculate various metrics representing different aspects of the landscape. The metrics for analysis attempts to understand object geometry. The metrics calculated, for these analyses, were the average and standard deviation for the area (AREA), the fractal dimension index (FRAC), and the perimeter area ratio (PARA). The number of patches (NP) was also included in each result. To take a more landscape centric approach, the area weighted mean was chosen over a simple average.

## Results

The following will present the empirical results with comparisons between the OOB error, the random forest classification, and segmentation discrepancy.

### Segmentation results

Like the classification results, Table 1 shows the PR segmentation results of the super objects, with and without the Gabor feature, with all features included (spectral, CHM, NDVI). Table 2 shows the PR segmentation results for the sub-objects. The inclusion of the Gabor feature for the super objects made very little difference (0.03 percentage points) in the segmentation results according to the PR metric. In the sub-object case, the inclusion of the Gabor feature greatly decreased segmentation performance.

**Table 1:** Out-of-bag error (super).

Feature	Out-of-bag error (%)	Pr %
Without gabor	16.15	21.65
With gabor	18.59	21.62

Out-of-bag error results from the random forest classifier and percentage of right segmented pixels (PR) in the whole image for the super-objects.

**Table 2:** Out-of-bag (Sub).

Feature	Out-of-bag error	Pr %
Without Gabor without hierarchy	22.53%	13.99%
With Gabor without hierarchy	23.01%	71.63%
Without Gabor with hierarchy	16.11%	13.50%
With Gabor with hierarchy	12.71%	69.50%

Our-of-bag error results from the random forest classifier and percentage of right segmented pixels (PR) in the whole image for the sub-objects.

### Classification results

Table 1 exhibits the OOB classification results from the random forest classifier of the super objects, with and without the Gabor feature, with all features included (spectral, CHM, NDVI). These results were used as the hierarchical features for the sub-objects. The sub-object’s OOB classification results can be viewed in Table 2. As shown, the only instance when the inclusion of the Gabor feature improved classification results, for both the super and sub-objects, was when the hierarchical feature was included. The hierarchal feature improved accuracy in both instances but was further improved when combined with the Gabor feature, resulting with the best performance of the four datasets.

### Landscape metrics

Results for the landscape metric analyses can be seen in Table 3. The differences between sub and super objects were as expected, with super objects having fewer patches than the sub-objects and area weighted mean being larger for the super objects except for sub-objects created with the Gabor features. The sub-objects created with the Gabor features had area averages that were greater than any other results, including the human segmented results, which is due to large continuous patches of wet forests. Additionally, these instances had the largest standard deviations in patch size. This indicates that there was a broad mixture of large and small patches.

**Table 3:** Landscape metrics.

	NP	AREA_AM	AREA_SD	FRAC_AM	FRAC_SD	PARA_AM	PARA_SD
Gab super	62,905	48.83	0.92	1.385	0.362	1382.58	16,912.97
Gab sub	87,198	157.36	1.40	1.345	0.365	1215.01	14,595.74
GAB sub Hier	87,225	155.03	1.39	1.342	0.366	1216.48	14,717.95
NDVI super	51,664	46.48	0.99	1.366	0.319	1166.51	16,133.02
NDVI sub	191,050	16.43	0.31	1.368	0.290	2044.08	12,614.72
NDVI sub Hier	189,106	16.89	0.31	1.367	0.290	2024.65	12,638.21
Control super	87,019	39.22	0.70	1.330	0.276	1096.12	9329.83
Control sub	104,549	20.33	0.46	1.230	0.259	1278.33	10,362.95

Calculated landscape metrics for human segmented objects and all instances of the automated segmented objects. *NP* number of patches, *AREA* square area of patch (m^2^), *FRAC* fractal dimension index of a patch, *PARA* perimeter area ratio of a patch, *AM* area weighted mean, *SD* standard deviation.

When observing the automated results for the super-objects, it appears that not including the Gabor features provides similar results to the human segmented objects. The only instance where the inclusion of Gabor features makes the segmentation similar to the human segmented objects is the number of patches, which can also be a measure of landscape fragmentation. Average area and both measures of edge or shape complexity (FRAC and PARA) both show that the exclusion of Gabor features cause segments to be more similar to the human segmented objects.

Similar observations can be made for the sub-objects. In most cases, instances where the Gabor feature was excluded resulted in similar landscape metrics to the human segmented objects. Furthermore, the inclusion of Gabor features had a higher number of similar patches as the human segmented instances for the sub-objects. The exclusion of Gabor features severely over fragment the landscape whereas the inclusion of Gabor features slightly under-fragmented it. It was observed that when the Gabor features were included within the segmentation, the areas classified as wet forest (a significant proportion of land cover in the study area) were delineated into large patches. When the Gabor features was not included, the wet forest was over fragmented which contributed to the large number of patches. A characteristic of the wet forest class in the study area is that they existed as large continuous chains and perhaps the first principal component did a good job at capturing the textural attribute of this class. In future studies, more principle components should be included on the chance that they can capture the textural attributes of the other classes better than the first principal component alone.

## Discussion

Our study yields valuable information to the inclusion of hierarchically organized vectors; it provides accuracy estimates for classified objects with and without the inclusion of hierarchal attributes. Of the identified papers that used hierarchical segmentation, few included hierarchical attributes in their object classification^18,49,50^ and only one included the accuracy estimates with and without the inclusion of hierarchal attributes^15^. Other studies used one segmentation scale to guide the segmentation results of the next finer or broader scale^16,17,19,51^. Antunes et al.^15^ report agreed with our results in that the inclusion of hierarchical attributes increased classification accuracies considerably. Laiberte et al.^50^, Laiberte et al.^18^, and Laiberte et al.^49^ supported our findings by stating that including hierarchical attributes visibly improved their results^50^.

Observing both variable indices, the Gini and mean decrease in accuracy index, each instance the hierarchical features were included as an attribute for the sub-objects, the hierarchical features were indicated as providing more predictive power relative to the other included features. This coincides with the increase in accuracy when these features were included and, therefore, does not provide sufficient evidence that hierarchical features introduce noise into the dataset but rather provide valuable predictive information. This is contrary to the results observed when Gabor features were included, in the random forest, for predicting super-objects. The mean decrease in accuracy index indicated a high predictive power for the Gabor features, and an increased OOB error.

Gabor features did not provide additional information for increasing classification accuracies or improve segmentation results according to the sub-objects’ PR metric. The super-objects’ PR metric decreased insignificantly when Gabor features were included in the segmentation step. The PR metrics for the sub-objects display a significant decrease in segmentation accuracy when Gabor features are included. Based on these results, Gabor features should not be included in the segmentation step of the GEOBIA process. According to these results, Gabor features should not be included as part of the training and classification unless hierarchical features are included.

It is unclear to the authors why the inclusion of Gabor features improved the classification results only in the instance when hierarchical features are included. According to the Gini index and mean decrease in accuracy, the random forest algorithm utilized the Gabor features slightly more when hierarchical features were included than when they were not suggesting that the Gabor features improved the classification. When Gabor features were included for the other sub-object datasets that did not include hierarchical features, these same indices showed that Gabor features were utilized very little by the random forest algorithm (in addition to decreasing their accuracy). Similar effects were found when Gabor features were included within a patch based land cover analysis^33^. It is suggested that further research is conducted to observe why Gabor features have the opposite effect on classification when hierarchical features are included.

Limitations of this analysis of the segmentation results are as follows. To begin, the metrics used to evaluate segmentation results are still being developed. Most segmentation evaluations within geography use discrepancy measures, based off a classified ground truth image^37,46,47,52,53^. These measures depend on the correct classification of the objects and heavily relies on the accuracy of the classifier rather than measuring the quality of boundaries created by an algorithm. One proposed method is to measure the distance between the boundaries of the ground truth images and those generated by the proposed algorithm.

Another limitation is that most empirical methods for segmentation evaluation are based on ground truth images that are generated by human subjects, who subjectively delineate image object boundaries. Human interpretation can be inconsistent, biased, and differ from person to person despite any expert status. The PR metric is also influenced by the correct classification of the objects. One reason object-based analysis is widely used is that it produces consistent, predictable, and reproducible results. Rather than relying on correctly classified pixels for segmentation evaluation, object-based image analysis should begin using distance to reference boundaries^54^. Additionally, most users conducting an object-based image analysis, to aid in decision-making process, do a considerable amount of post-processing (i.e. dissolving small segments and holes, smoothing, merging) which could cause the PR metric, and other metrics to observe segmentation results, to change.

Natural multipart landscapes are complicated systems that have spatially interconnected parts that influence one another across space and scales. Not utilizing this information (i.e. pixel-based classification) or ignoring to identify spatial or hierarchical relationships does not fully exploit the information that can be obtained from delineation and classification of objects. Our results provided further support that including hierarchical structure to objects offers contextual information that can increase classification accuracy beyond what is provided by texture and spectral alone.

## Data Availability

Please contact the authors for data and material related to this work.
